# Physicochemical properties of malted finger millet (*Eleusine coracana*) and pearl millet (*Pennisetum glaucum*)

**DOI:** 10.1002/fsn3.816

**Published:** 2018-10-11

**Authors:** Joseph O. Owheruo, Beatrice O. T. Ifesan, Ayodele O. Kolawole

**Affiliations:** ^1^ Department of Food Science and Technology Delta State Polytechnic Ozoro Delta State Nigeria; ^2^ Department of Food Science and Technology Federal University of Technology Akure Akure Ondo State Nigeria; ^3^ Department of Biochemistry Federal University of Technology Akure Akure Ondo State Nigeria

**Keywords:** antioxidant activity, germination, millet, physicochemical, phytochemicals

## Abstract

Germinated and raw finger millet (*Eleusine coracana*) and pearl millet (*Pennisetum glaucum*) were investigated for their physicochemical (pH, total titratable acidity (TTA), proximate, mineral analysis), phytochemical, and antioxidant properties. The results showed that there were decreases in pH (8.50–7.60) with a corresponding increase in TTA (0.0038–0.18 g/L) during germination of the millets. Proximate composition of the millets revealed slight increases in protein (7.61%–7.81%; 10.57%–11.87%) and crude fiber (5.54%–8.81%; 1.07%–2.55%) with reductions in fat (3.84%–2.73%; 7.69%–2.30%) after germination for finger and pearl millet, respectively. The millets were found to be rich sources of minerals, which include magnesium (1,028.42–1,763.50 ppm), calcium (36.42–4,158.40 ppm), sodium (150.00–510.00 ppm), potassium (470.00–4,500 ppm), zinc (20.00–40.00 ppm), and iron (66.00–121.00 ppm) which either decreased or increased with germination. The results of the phytochemical composition revealed that during germination, alkaloid (36.03–74.53 mg/g) and saponin (4.46–31.91 mg/g) contents were found to increase while there were reductions in tannin (0.88–1.64 mg/g) and phytate (7.00–17.72 mg/g) content of the flour. For finger millet, DPPH ranged from 70.00% to 72.14% and pearl millet (49.95%–64.01%), while for FRAP, pearl millet (53.69–53.76 mg/g) demonstrated better activity compared to finger millet (46.91–53.54 mg/g). Findings from this work may suggest that further studies should be carried out on germinated finger and pearl millets to examine their abilities to serve as functional foods.

## Abstract 2

Germinated and raw finger (*Eleusine coracana*) and pearl millet (*Pennisetum glaucum*) were investigated for their physicochemical (pH, total titratable acidity [TTA], proximate, mineral analysis), phytochemical, and antioxidant properties.

## 1 INTRODUCTION

The use of cereals in food preparation is on the increase due to the fact that they contain phytochemicals and dietary fibers that possess good nutritional benefits (Lansakaraa et al., 2016). Previous researches have revealed that regular consumption of cereals and their products can protect the consumer against several diseases (McKeown, Meigs, Liu, Wilson, & Jacques, 2002).

Pearl millet (*Pennisetum glaucum*) is one of the most important drought‐tolerant crops of the tropics (Zakari, Hassan, & Abbo, 2010). United States Department of Agriculture reported that Nigeria contributes about 6.7 million tons of pearl millet to the World production (USDA, 2005). Despite the numerous involvement of millet grains in the production of value‐added foods in developing countries, it is still found to be underutilized. Pearl millet is said to be richer than corn in essential amino acid and minerals (Abdalla, El Tinay, Mohamed, & Abdalla, 1998).

Finger millet was found to compete favorably with barley, rye, and oats and able to deliver expected nutritional requirement for growing children, pregnant women, aged, and invalid (Satish, Ceasar, & Ramesh, 2017). The fiber is bulky and digested slowly, while the consumer is satisfied with fewer calories and this prevents consumption of excess calories. Several researchers have shown that finger millet exhibited numerous health benefits (Chandra, Chandra, & Pallavi, 2016; Devi, Vijayabharathi, Sabathyabama, Malleshi, & Priyadarisin, 2014; Lakshmi & Sumathi, 2002; Satish, Rency, & Ramesh, 2018).

Germination is a processing method that improves the nutritive value of cereals (Ayo, Ayo, Popoola, Omosebi, & Joseph, 2014) legumes (Egounlety & Aworh, 2003; Olagunju & Ifesan, 2013) with increases in methionine and cysteine content (Mibithi‐Mwikya, Camp, Yiru, & Huyghebaert, 2000). Several studies have demonstrated antioxidant (Chandrasekara & Shahidi, 2011; Veenashri & Muralikrishna, 2011) and antimicrobial properties of finger millet (Chethan & Malleshi, 2007; Varsha, Urooj, & Malleshi, 2009). The outer layer of the grain contains dietary fiber, minerals, phenols, and vitamins which offer nutritional and health benefits (Antony, Sripriya & Chandra, 1996). Malting of millet and sorghum has been a traditional practice in Africa to produce infant food, lactic acid, and alcoholic fermented beverages (Issoufou, Mahamadou, & Guo‐Wei, 2013). The aim of this work therefore is to examine the effect of germination on the physicochemical properties of finger millet and pearl millet flour. This study may provide information on the possible application of malted millet flour as functional food component in the food industry.

## 2 MATERIALS AND METHODS

### 2.1 Source of materials

Millet samples (*Eleusine coracana* and *P. glaucum)* were purchased from Ganawuri Market, Jos, Plateau State, Nigeria, in the month of January 2016. All chemicals used for the analyses were of analytical grade.

### 2.2 Processing of raw and germinated millet flour

Millet was sorted and oven dried (Model No. DHG‐9101 ISA) at 60°C for 8 hr. The dried grain was milled in an attrition mill, packed in polyethylene nylon, and stored at room temperature prior to further analyses as raw millet flour. Sprouting was achieved by spreading millet on a moistened jute sack and left to germinate at room temperature for 3 days. The germinated grains were washed with distilled water, drained, and oven dried (Model No. DHG‐9101 ISA) at 60°C. The dried grains were milled, packaged in an airtight container, and stored under room temperature prior to analyses (Badau, Nkama, & Jideani, 2005).

### 2.3 Determination of pH and total titratable acidity of pearl and finger millet flour

The pH of millet flour sample was measured by mixing 10 g of the germinated millet with 90 ml of distilled water. This was then ground with mortar and pestle to allow proper dissolution and the pH was determined.

Total titratable acidity was determined following the method of Association of Official Analytical Chemists (AOAC, 2005 2005). Ten milliliter of aliquot of millet sample was mixed with two drops of phenolphthalein indicator in a test tube and was thoroughly shaken. The mixture was titrated against 0.1 M NAOH until there was a change in color to persistent pink end point and acidity was calculated (James, 1999).

### 2.4 Determination of proximate composition of millet flour

Proximate composition of raw and germinated flour was determined according to the method of Association of Official Analytical Chemists (AOAC) (2005 2005).

### 2.5 Millet flour mineral content determination

The mineral composition (magnesium, calcium, sodium, potassium, zinc, and iron) of the millet flour samples was determined using Association of Official Analytical Chemists (AOAC) (2005 2005) methods.

### 2.6 Determination of phytochemical properties of millet flour

The method of Makkar and Goodchild (1996) was used to determine the tannin content of millet. A sample of millet was weighed, mixed with 10 ml of 70% aqueous acetone, properly covered, and placed in an ice bath shaker at room temperature for 2 hr. After this, the solution was centrifuged and the supernatant was stored in ice. About 0.2 ml of the solution was introduced into the test tube that contained 0.8 ml of distilled water, while 0.5 ml Folin–Ciocalteu reagent and 2.5 ml of 20% Na_2_CO_3_ were added. The mixture was vortex and allowed to incubate for 40 min at room temperature, while absorbance was read at 725 nm.

Phytate content of millet sample was determined following Wheeler and Ferrel (1971). Four grams of millet was soaked in 100 ml of 2% HCl for 3 hr and then filtered through a No. 1 Whatman filter paper. Twenty‐five milliliter of the filtrate was mixed with 5 ml of 0.3% ammonium thiocyanate solution as indicator after which 53.5 ml of distilled water was added to give it the proper acidity. This was titrated against standard iron (III) chloride solution that contained about 0.00195 g of iron per milliliter until a brownish yellow color persist for 5 min.

Saponin content of millet flour was determined according to Brunner (1984). Two grams of millet flour was weighed into 100 ml of isobutyl alcohol. The mixture was placed in the shaker for 5 hr to ensure thorough mixing. It was then filtered with No. 1 Whatman filter paper and 40% saturated solution of magnesium carbonate was added to it. The mixture obtained was again filtered. Two milliliter of 5% iron (III) chloride (FeCl_3_) solution was added to the 1 ml of the filtrate inside a 50‐ml volumetric flask and made up to the mark with distilled water. It was allowed to stand for 30 min for color development, while the absorbance was read against the blank at 380 nm.

To determine the alkaloid content of millet flour sample, 5 g of millet flour was weighed into 200 ml of 10% ethanol‐acetic acid and was allowed to stand for 4 min. This was filtered and concentrated ammonium hydroxide was added in drops to the filtrate until it formed precipitate. This was filtered and the extract was concentrated on a water bath to one quarter of the original volume. Concentrated ammonium hydroxide was added drop wise to the extract until the precipitation was completed. The whole solution was allowed to settle, the precipitate was collected, washed with dilute ammonium hydroxide, and then filtered. The residue was the alkaloid, which was dried and weighed (Harborne, 1973).

### 2.7 Determination of antioxidant properties of millet flour

Total phenol content of millet flour sample was determined following Singleton, Orthofer, and Lamuela‐Raventos (1999). About 0.2 ml of the extract from millet flour was mixed with 2.5 ml of 10% Folin–Ciocalteu reagent and 2 ml of 7.5% sodium carbonate. The mixture was placed in an incubator for 40 min at 45°C, while the absorbance was read at 700 nm with the spectrophotometer. Gallic acid was used as the standard.

About 0.2 ml of the millet flour extract was mixed with 0.3 ml of 5% NaNO_3_. After 5 min, 0.6 ml of 10% AlCl_3_ was introduced and later 2 ml of 1 M NaOH and 2.1 ml of distilled water was added to the mixture. Absorbance was read at 510 nm against the reagent blank, and total flavonoid content was expressed as mg/g (Bao, Cai, Sun, Wang, & Corke, 2005).

The method of Pulido, Bravo, and Saura‐Calixto (2000) was used to determine a ferric reducing property of millet flour. About 0.25 ml of millet flour extract was mixed with an equal volume of 200 mM of sodium phosphate buffer and 1% potassium ferricyanide. The mixture was allowed to incubate at 50 °C for 20 min after which 0.25 ml of 10% TCA was added and centrifuged at 458.38 *g*. One milliliter of the supernatant was then mixed with 1 ml of distilled water and 0.1% of FeCl_3_, while the absorbance was read at 700 nm.

The free radical scavenging ability of millet flour extract against DPPH was carried out using Gyamfi, Yonamine, and Aaniya (1999) method. One milliliter of the extract was mixed with 1 ml of 0.4 mM methanolic solution of the DPPH and the mixture was left in the dark for 30 min after which the absorbance was read at 516 nm.

### 2.8 Statistical analysis

The mean and standard error of means of the triplicate analyses were calculated. The analysis of variance (ANOVA) was performed to determine significant differences between the means, while the means were separated using the new Duncan multiple range test.

## 3 RESULTS AND DISCUSSION

### 3.1 pH and total titratable acidity of germinated millet

The result in Table 1 shows that pH decreased from 8.50 at 0 hr to 7.60 for pearl millet and 7.90 for finger millet, while TTA increased from 0.0038 to 0.18 g/L during germination. The decrease in pH and increase in TTA might be due to degradation of some complex organic molecules such as lipids, phytin, and protein to simpler compounds. Thus, the increase in acidity as germination took place could be related to the rate at which complex compounds were hydrolyzed (Gernah, Ariahn, & Ingbian, 2011). The result obtained in this present study is in agreement with the findings of Adeyemo, Olayode, and Odutuga (1992) who reported reduction in pH and increased acidity as sprouting progressed in maize. The pH value of the millets observed in this study (8.30–7.60) is higher than the value reported for maize (6.80–6.20) by Gernah et al. (2011). It was reported that pearl millet could raise the hydrogen ion content of the stomach to an alkaline state and thus reduced the occurrence of ulcer (Shweta, 2015).

**Table 1:** pH and titratable acid during germination

Days (hr)	African finger millet		Pearl millet	
	pH	TTA	pH	TTA
0	8.50 ± 0.02^a^	0.038 ± 0.01^c^	8.30 ± 0.01^a^	0.14 ± 0.01^a^
24	7.50 ± 0.01^b^	0.036 ± 0.01^c^	7.50 ± 0.01^b^	0.16 ± 0.01^a^
48	7.20 ± 0.01^b^	0.04 ± 0.03^b^	7.70 ± 0.01^b^	0.17 ± 0.02^a^
72	6.20 ± 0.01^c^	0.17 ± 0.01^a^	6.10 ± 0.01^c^	0.18 ± 0.01^a^

*Note*

Values represent mean of triplicate ± standard deviation. Mean with the same letter in a column is not significantly different (*p* < 0.05).

### 3.2 Proximate composition of raw and germinated millet

The moisture content of millet ranged from 2.16% to 4.50%, with the highest value from raw pearl millet flour (Table 2). Storage periods of food products can be extended when the moisture content is low as this will prevent microbial infestation (Alozie, Iyam, Lawal, Udofia, & Ani, 2009). The result of the proximate composition revealed that protein and crude fiber of millets increased during germination. The protein content was from 7.61% to 11.87% with a significant increase in the pearl millet protein. This observation agrees with other scientific findings that sprouting brought about an improvement in the nutritional quality of food products particularly protein content (Alozie et al., 2009; Enujiugha, Badejo, Iyiola, & Oluwamukomi, 2003). The increase in protein content of the germinated millet may be as a result of the formation of enzymes or an encompassing change following degradation of other constituents (Ijarotimi, 2012). Finger millet flour possesses a higher value for crude fiber (5.54%–8.81%) than that of pearl millet (1.07%–2.55%). During germination sugar in the seed is usually used up leaving only the fibrous seed, and this might be the reason for the increase in fiber content (Ikenebomah, Kok, & Ingram, 1986). Similar observations have been reported by Ali, El Tinay, and Abdalla (2003) in pearl millet (2.8%) and Gunashree, Selva, Roobini, and Venkateswaran (2014) in ragi and wheat. In addition, pearl millet is a rich source of dietary fiber and micronutrients (Shegal & Kwatra, 2006). The fat content of germinated finger millet (3.84%–2.73%) and pearl millet (7.69%–2.30%) is low. Similar results of 7.8% and 5.1% crude fat, respectively, have been reported in pearl millet (Ali et al., 2003; Taylor, 2004). It was observed that carbohydrate in finger millet (78.08%–75.70%) decreased, while that of pearl millet (74.41%–79.09%) increased significantly with germination and both are higher than 63.2% reported for pearl millet (Ali et al., 2003).

**Table 2:** Proximate compositions of raw and malted millet flours

Major nutrients (g/100 g)	Raw finger millet flour	Germinated finger millet flour	Raw pearl millet flour	Germinated pearl millet flour
Moisture	3.10 ± 0.53^a^	3.14 ± 0.56^a^	4.50 ± 0.49^b^	2.16 ± 0.5^a^
Protein	7.61 ± 0.01^a^	7.81 ± 014^a^	10.57 ± 0.26^b^	11.87 ± 0.40^c^
Ash	1.84 ± 0.59^a^	1.80 ± 0.27^a^	2.00 ± 0.02^a^	2.04 ± 0.51^a^
Fiber	5.54 ± 0.03^c^	8.81 ± 0.01^d^	1.07 ± 0.39^a^	2.55 ± 0.06^b^
Fat	3.84 ± 0.55^b^	2.73 ± 0.25^a^	7.69 ± 0.24^c^	2.30 ± 0.26^a^
Carbohydrate	78.08 ± 052^c^	75.70 ± 0.44^b^	74.41 ± 0.64^a^	79.07 ± 0.96^c^

*Note*

Values represent mean of triplicate ± standard deviation. Mean with the same letter in a row is not significantly different (*p* < 0.05).

### 3.3 Mineral composition of raw and germinated millet flour

Magnesium content of pearl millet was observed to increase during germination from 1,028.60 to 1,070.60 ppm, while it decreased in the finger millet from 1,763.50 to 1,616.60 ppm (Table 3). Calcium content of raw finger millet flour increased from 822.40 to 4,158.40 ppm after germination while that of raw pearl millet (36.92–92.42 ppm) was found to decrease. This result suggests that the germinated millet flour may be a good source of calcium. Ijarotimi (2012) reported an increase in calcium content of wheat flour after germination. Sodium content of pearl millet (230.00–380.00 ppm) and potassium (470.00–2,295.00 ppm) of finger millet were also found to increase during germination. Sodium and potassium are important in the diet due to the roles they perform in blood pressure regulation (Yoshimura, Takahashin, & Nakanishi, 1991). The ratio of Na/K of germinated flour samples was 0.07 for finger millet and 0.13 for pearl millet, and it is below 1.0 recommended by National Research Council (NRC, 1989). Millet flour may be considered as a diet to regulate blood pressure and nerve functions in the body. Zinc content of pearl millet (39.00–40.00 ppm) was significantly higher than that of finger millet (20.00–30.00 ppm). This result is contrary to the previous report where there was an increase in zinc content after germination of popcorn (Ijarotimi & Keshinro, 2011). Zinc is a multifunctional nutrient needed in glucose and lipid metabolism, hormone functionality, and wound healing (Obiajunwa, Adebiyi, & Omode, 2005).

**Table 3:** Mineral Compositions (ppm) of raw and malted millet flours

Minerals	Raw finger millet flour	Germinated finger millet flour	Raw pearl millet flour	Germinated pearl millet flour
Magnesium	1,763.5 ± 0.9^d^	1,616.6 ± 1.0^c^	1,028.6 ± 1.0^a^	1,070.6 ± 1.0^b^
Calcium	822.4 ± 1.0^c^	4,158.4 ± 1.0^d^	36.42 ± 0.6^a^	92.42 ± 1.0^b^
Sodium	510.0 ± 1.0^d^	150.0 ± 1.0^a^	230.0 ± 1.0^b^	380.0 ± 1.0^c^
Potassium	470.0 ± 1.0^a^	2,295.0 ± 1.0^b^	4,500.0 ± 1.0^d^	2,999.7 ± 0.5^c^
Zinc	30.0 ± 1.0^b^	20.0 ± 1.0^a^	40.0 ± 1.0^c^	39.0 ± 1.0^c^
Iron	91.0 ± 1.0^c^	74.0 ± 1.0^b^	121.0 ± 1.0^d^	66.0 ± 1.0^a^

*Note*

Values represent mean of triplicate ± standard deviation. Mean with the same letter in a row is not significantly different (*p* < 0.05).

### 3.4 Phytochemical properties of raw and germinated millet

The alkaloid content of the millet flour increased during germination while that of pearl millet (49.50%–74.53%) was higher than finger millet (36.03%–68.44%) (Table 4). Alkaloids are active components of esthetics, sedative, stimulants, relaxants, and tranquilizers. Alkaloids are employed in medicine because they can act quickly on specific areas of the nervous system. The result may be explained that germinated millet can possess medicinal property.

**Table 4:** Phytochemical properties of raw and malted millet flours

Phytochemical	Raw finger millet flour	Germinated finger millet flour	Raw pearl millet flour	Germinated pearl millet flour
Alkaloid (%)	3.60 ± 0.07^a^	6.84 ± 1.29^c^	4.95 ± 0.21^b^	7.45 ± 0.07^d^
Tannin (mg/g)	1.64 ± 0.01^d^	1.50 ± 0.01^c^	0.88 ± 0.28^a^	1.02 ± 0.01^b^
Saponin (mg/g)	1.80 ± 0.01^c^	3.19 ± 0.13^d^	0.45 ± 0.80^a^	0.86 ± 0.39^b^
Phytate (mg/g)	14.02 ± 0.01^c^	10.72 ± 0.01^b^	17.72 ± 0.59^d^	7.00 ± 0.58^a^

*Note*

Values represent mean of duplicate ± standard deviation. Mean with the same letter in a row is not significantly different (*p* < 0.05).

Tannin content in the raw millet flour (1.64 mg/g; 1.02 mg/g) was observed to reduce significantly during germination (1.50 mg/g; 0.88 mg/g) for finger and pearl millet, respectively. Tannins have been reported to lower digestibility of most nutrients, especially protein (Ali et al., 2003). The observed reduction in tannin content of germinated seeds may be as a result of tannin binding to proteins and enzymes and not due to loss or degradation of tannin (Mibithi‐Mwikya et al., 2000). However, the decrease in tannin content during germination has been explained as leaching of tannin from the sprouting mass and decreased activity of polyphenoloxidase and other metabolic enzymes (Shimelis & Rakshit, 2008).

Result further showed that phytate content of finger millet (14.02–10.72 mg/g) and pearl millet (17.72–7.00 mg/g) decreased after germination. Processing methods such as soaking, fermentation, and germination have been earlier reported to reduce phytate content for some seeds (Shimelis & Rakshit, 2008).

Saponin content increased from 18.01 to 31.91 mg/g in finger millet and 4.46 to 8.64 mg/g in pearl millet flour. This was probably as a result of the displacement of stored phytochemical from the sprouts (Rupasinghe et al., 2003). Saponin is thought to be beneficial to human in the function of several organ systems and treating a variety of diseases. Ingestion of saponin has been linked with a decrease in overall blood cholesterol. Abundance of phytochemicals in millets may enhance their nutraceutical potentials, thereby making them reliable source of functional foods.

### 3.5 Antioxidant properties of raw and germinated millet

Total flavonoid content of finger millet was found to decrease (1.4 to 1.09 mg/g), while there was no change in that of pearl millet (0.91 mg/g) during germination (Table 5). The presence of flavonoid, a phenolic compound in millet flour, may contribute to the health potential of the flour. Flavonoid has been reported to exhibit antioxidant activity (Middleton, Kandaswami, & Theoharides, 2000).

**Table 5:** Antioxidant properties of raw and malted millet flours

Antioxidant	Raw finger millet flour	Germinated finger millet flour	Raw pearl millet flour	Germinated pearl millet flour
DPPH ((%)	70.0 ± 0.79^c^	72.14 ± 0.64^c^	49.95 ± 0.43^a^	64.01 ± 0.65^b^
FRAP (mg/g)	46.91 ± 0.16^a^	53.54 ± 0.04^b^	53.76 ± 0.01^b^	53.69 ± 0.01^b^
Flavonoid (mg/g)	1.44 ± 0.04^c^	1.09 ± 0.01^b^	0.91 ± 0.14^a^	0.91^a^ ± 0.05
Phenol (mg/g)	1.57 ± 0.04^b^	5.70 ± 0.02^d^	1.45 ± 0.01^a^	4.27^c^ ± 0.02

*Note*

Values represent mean of duplicate ± standard deviation. Mean with the same letter in a row is not significantly different (*p* < 0.05).

The phenol in the flour samples was 1.57–5.70 mg/g and 1.45–4.27 mg/g for finger millet and pearl millet, respectively. Dhan and Gange (2012) reported a similar observation in finger millet. According to Maillard and Berset (1995), increase in phenol content during germination may be due to enzymatic release of bound phenolic compound. Phenols may play a role in antioxidative potential of grains and contribute to extension of shelf‐life of cereal products (Banerjee, Sanjay, Chethan, & Malleshi, 2012).

Free radical scavenging activity (DPPH) in the raw samples (70.0%; 49.95%) increased to 72.14% and 64.01%, respectively, in finger millet and pearl millet after germination (Table 5).

Ferric reducing power (FRAP) of finger millet flour (46.91–53.54 mg/g) increased after germination, while there was little or no difference in pearl millet flour (53.76–53.69 mg/g). Previous studies have shown that the reducing ability of a substance may explain its potential antioxidant activity (Adesegun, Elechi, & Coker, 2008; Sofidiya, Odukayo, Familoni, & Inya‐Agha, 2006). The result from this present study showed that finger and pearl millet possess the ability to scavenge free radicals and therefore can be employed as a source of antioxidant to prevent accumulation of unwanted substances in the system (Odusola, Ilesanmi, & Akinloye, 2013).

## 4 CONCLUSION

Germination brought about an appreciable increase in protein, fiber, and some minerals in the millet samples. In addition, malted millets were found to possess certain phytochemicals which may be responsible for the antioxidant properties demonstrated. Therefore, further research is needed to investigate the potential of the germinated millets to serve as functional foods.

## CONFLICT OF INTEREST

The authors have declared no conflict of interests.
